# Ecosystem Services in Conservation Planning: Targeted Benefits vs. Co-Benefits or Costs?

**DOI:** 10.1371/journal.pone.0024378

**Published:** 2011-09-06

**Authors:** Kai M. A. Chan, Lara Hoshizaki, Brian Klinkenberg

**Affiliations:** 1 Institute for Resources, Environment and Sustainability, University of British Columbia, Vancouver, Canada; 2 Department of Geography, University of British Columbia, Vancouver, Canada; University of California, Berkeley, United States of America

## Abstract

There is growing support for characterizing ecosystem services in order to link conservation and human well-being. However, few studies have explicitly included ecosystem services within systematic conservation planning, and those that have follow two fundamentally different approaches: ecosystem services as intrinsically-important targeted benefits vs. substitutable co-benefits. We present a first comparison of these two approaches in a case study in the Central Interior of British Columbia. We calculated and mapped economic values for carbon storage, timber production, and recreational angling using a geographical information system (GIS). These ‘marginal’ values represent the difference in service-provision between conservation and managed forestry as land uses. We compared two approaches to including ecosystem services in the site-selection software Marxan: as *Targeted Benefits*, and as *Co-Benefits/Costs* (in Marxan's cost function); we also compared these approaches with a *Hybrid* approach (carbon and angling as targeted benefits, timber as an opportunity cost). For this analysis, the Co-Benefit/Cost approach yielded a less costly reserve network than the Hybrid approach (1.6% cheaper). Including timber harvest as an opportunity cost in the cost function resulted in a reserve network that achieved targets equivalently, but at 15% lower total cost. We found counter-intuitive results for conservation: conservation-compatible services (carbon, angling) were positively correlated with each other and biodiversity, whereas the conservation-incompatible service (timber) was negatively correlated with all other networks. Our findings suggest that including ecosystem services within a conservation plan may be most cost-effective when they are represented as substitutable co-benefits/costs, rather than as targeted benefits. By explicitly valuing the costs and benefits associated with services, we may be able to achieve meaningful biodiversity conservation at lower cost and with greater co-benefits.

## Introduction

Although the area of land protected has expanded considerably across the planet [1], such areas are alone insufficient to protect more than a small fraction of biodiversity [2], [3], [4], [5]. But, in this crowded world, it is increasingly difficult to justify conservation for biodiversity's sake without also demonstrating benefits for people. The concept of ecosystem services (the provision of benefits by ecosystems for people, directly and indirectly) offers a framework for characterizing and communicating the numerous benefits of conservation for people, such as food provision, water purification, and flood mitigation [2], [6]. Proponents of ecosystem services hope that this concept will expand conservation activities and funding for them [7] while continuing to benefit people [8], [9].

Ecosystem-based approaches to management are rapidly gaining momentum, with governments across the world requiring the simultaneous consideration of ecological sustainability and the flow of multiple benefits to people (ecosystem services) from ecosystems [10], [11]. While much is known about individual ecosystem services (e.g., pollination [12], [13] and carbon sequestration as a means of climate regulation [14]), little is known about the distributions of multiple services alongside conservation priorities in landscapes [15] or their compatibility with biodiversity conservation. Recent research suggests that areas with high levels of biodiversity are not necessarily the areas that might be prioritized for ecosystem services [16], [17], [18], [19].

Biodiversity conservation is often a catalyst for ecosystem-based approaches to management. Yet, conservation planning lacks an established means to measure the full extent of costs and benefits associated with alternative conservation plans, for people. Because ecosystem services can be important co-benefits or opportunity costs of conservation, there has been considerable interest in ecosystem services in planning [20]. But in the vast majority of cases the integration of services has been merely through biodiversity patterns or ecological processes that are assumed to be relevant for services [20]—there is urgent need for research that investigates the advantages and disadvantages of alternative frameworks for planning for ecosystem services.

As the migration toward ecosystem services-based approaches for management increases, two key questions must be answered: (1) how well do biodiversity and ecosystem services correlate across space? And, given imperfect correlations, (2) how can we use existing planning tools to most effectively prioritize for a range of management considerations within a particular landscape and on a constrained budget? A central challenge in coupling ecosystem services research and conservation planning is that many efforts to value services [e.g., the biome–wide average value of all services provided by 1 hectare, as in—21] have no clear connection to decision-making [22]. Because planning requires knowledge of what might be lost or gained due to realistic changes [e.g.], [ the value of changed service–provision associated with change in management of 1 hectare, recognizing that services will often not be lost completely—8,23]—our approach is to explicitly calculate the ecosystem-service consequences of conservation at the scale of each planning unit.

To integrate ecosystem services into conservation planning, it is helpful to develop frameworks for marginal valuation compatible with the prevailing tools of reserve-design, such as Marxan [24]. Given the abundant popularity of Marxan with conservation practitioners and the importance of implementing conservation on the ground [25], we believe that using this approach has value, despite its limitations [16]. In the first published integration of services in Marxan, Chan et al. [16] considered differences in service provision between conservation and development in the central coast of California. A more significant challenge is posed by contexts in which primary alternative land-uses change the provision of services in more nuanced ways [e.g., lessening but not eliminating carbon storage—26]. In this study, we address this issue by calculating the difference in the value of services across conservation and timber harvesting land-use scenarios.

There is no clear approach for integrating ecosystem services explicitly into conservation planning. In conservation planning, species, communities, and ecosystems are benefits for which ‘targets’ are expressed [27], [28]. Reserve-design algorithms combine this information on benefits with a ‘cost surface’ [24] to specify protection of a network of places to meet the quantitative targets for these benefits [29]. Ecosystem services have been treated previously as ‘Targeted Benefits’ to be protected while minimizing costs [16]; and fishing has been incorporated as an opportunity cost in marine conservation planning analyses [our ‘Co-Benefit/Cost’ approach—30]. The Targeted Benefit approach includes services as *intrinsically important* within a framework of *cost-minimization*, whereas the Co-Benefit/Cost approach includes them as *substitutable* in a framework of *net-benefit-maximization*—a critical philosophical difference (see Discussion). Here, we expand the Benefit/Cost approach—including multiple ecosystem service values for the first time within the cost function of Marxan—and offer a first comparison with the Targeted Benefit approach. We also combine these two approaches by including biodiversity-congruent services (recreational angling and carbon storage) as targeted benefits and incongruent services (timber production) as costs (our ‘Hybrid’ approach) (Table 1).

**Table 1:** Scenarios examined in the current analysis, based on the biodiversity and or ecosystem services included and the approach adopted for each.

Scenario	Biodiversity	Recreational Angling	Carbon Storage	Timber Production
**Biodiversity**	Targeted	-	-	-
**Angling**	*-*	Targeted	-	-
**Carbon**	*-*	-	Targeted	-
**Timber**	*-*	-	-	Targeted
**BD + ES Targeted Benefits**	Targeted	Targeted	Targeted	Targeted
**BD + ES Hybrid (A, B)**	Targeted	Targeted	Targeted	Opportunity Cost
**BD + ES Co-Benefit**	Targeted	Co-benefit	Co-benefit	-
**BD + ES Co-Benefit/Cost**	Targeted	Co-benefit	Co-benefit	Opportunity Cost

Any ecosystem service could be included in conservation planning either as a *Targeted benefit* to be protected at a particular level (subject to cost constraints, with costs being minimized), or as a *Co-Benefit/Cost* to be maximized/minimized. BD  =  biodiversity; ES  =  ecosystem services. Parameters were adjusted to allow comparability of *BD + ES Targeted Benefits* with *BD + ES Hybrid A*, and of *BD + Hybrid B* with *BD + ES Co-Benefit/Cost*.

**Figure 1:**
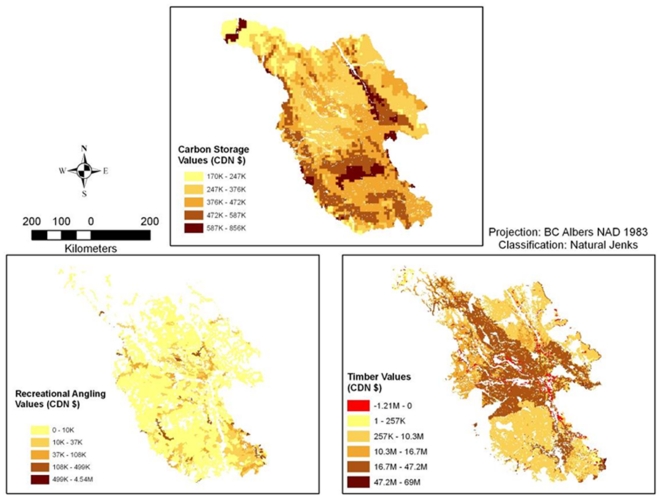
Map of study area. The study area is comprised of the Sub-boreal and Central Interior Eco-provinces in British Columbia, Canada.

Inherent in our approach is recognition of a fact often glossed over in conservation literature and rhetoric: activities that realize the benefits of ecosystem services (e.g., harvest to realize benefits of timber production) will frequently be at odds with biodiversity conservation. But even in such cases of incompatibility, there may be great gains in conservation efficiency by including these services in conservation planning as opportunity costs [30], [31]. In this paper we illustrate the inclusion of ecosystem services in a conservation plan in the Central Interior region of British Columbia (BC), Canada (Fig. 1), an ecoregional assessment of the Nature Conservancy of Canada (NCC) [32]. This assessment is a coarse-scale analysis of general areas meriting protection of biodiversity and ecosystem services, not a pinpointing of particular sites and conservation actions, as appropriate at finer scales [e.g., 33]. Our analysis focuses principally on the different representation of benefits and costs, and does not account for spatially variable threats. Accordingly, our ecosystem service layers are coarse-scale characterizations that do not reflect possible nuances in the management of such services.

We present spatially explicit, marginal economic values of three ecosystem services (carbon storage, timber production, and the provision of recreational angling opportunities) and integrate them in the Targeted Benefit, Co-Benefit/Cost, and Hybrid approaches to answer the two key questions posed above. Carbon storage refers to the carbon stored in above and below ground biomass as well as soil [34]. If the forests in the study area were to be harvested, rather than conserved, a portion of the carbon stored in the landscape currently would be released into the atmosphere and contribute to climate change. Conversely, a conserved landscape would contribute to the global service of climate regulation, a tradeoff between services [35]. The realization of benefits from timber production depends upon harvest, which as noted above is the primary threat to biodiversity in the region. The provision of recreational angling opportunities depends on several aspects of the surrounding landscape [36]. We assumed that timber harvesting increases sedimentation in streams due to soil erosion—a simplistic assumption only appropriate for the coarse scale of our planning exercise (see Materials and Methods). This sedimentation leads to the eventual degradation of fish habitat, thereby putting recreational angling at risk [37]. By protecting an area of land from timber harvesting, we may also protect instream habitat and recreational fishing species.

We chose to investigate these three services based on an informal survey of the NCC Central Interior team of experts, and in consideration of available data. All three services were chosen based on the demonstration of changes in value due to timber harvesting (the greatest threat to conservation in the area) and their importance to local beneficiaries. The analysis consisted of two primary objectives: (1) obtaining an economic valuation of ecosystem services and mapping these services with biodiversity features and (2), the inclusion of these values for a set of Marxan analyses, in part to compare the efficiency of the Targeted Benefit, Co-Benefit/Cost, and Hybrid approaches. The goal of this work is to guide conservation thinking in the study region regarding the extent to which recreational angling, timber harvest, and carbon storage offer opportunities or obstacles for conservation, the places where these opportunities/obstacles arise most strongly, and the means by which systematic conservation planning might account for these services.

## Results

### Ecosystem service values

**Figure 2:**
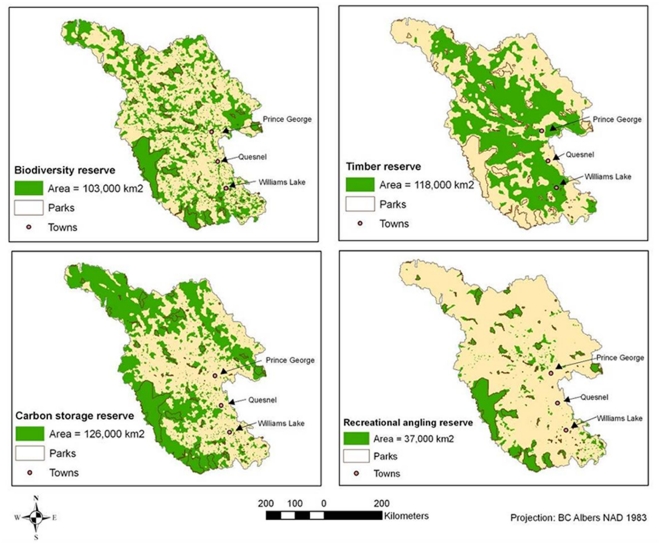
The economic values of ecosystem services in the Central Interior, BC. Values represent net present values of the difference between conservation and timber harvesting land management scenarios (in CDN $ per 500-ha planning unit). Study area is trimmed from surrounding land (Fig. 1).

The estimated net present value of changes in ecosystem services associated with a difference in management between timber harvesting and conservation are highly variable within and between services (Fig. 2). Values per 500-ha planning unit are considerably higher for timber harvesting (an estimate of net revenues) than for recreational angling or carbon storage (estimates of social benefits from non-market valuation); maximum values are higher for angling than carbon, but these high-angling values are limited to a small portion of the study area, whereas carbon values are more evenly distributed (Fig. 2).

It is unsurprising that timber harvest values dominate the other two services modeled, given that managed forestry is the prevailing land use/land cover in the region. A small but notable fraction of the landscape is characterized by negative (red) or negligible (light yellow) timber values, meaning that harvest-operation costs are projected to outweigh benefits (Fig. 2). Such low or negative values generally occur in areas with steep slopes, since timber harvest is more costly and difficult in such areas [38].

### “Best” solution reserve networks

**Figure 3:**
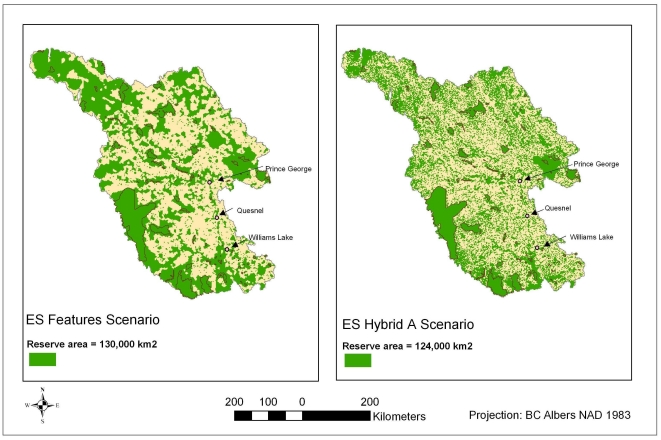
“Best” reserve network solutions for Biodiversity, Timber, Carbon and Angling scenarios. All three networks resulted from Marxan runs with road (‘suitability’) index scores as a cost surface. The “best” solution is the one solution out of 500 that captures all targets at the lowest total cost. Existing parks and protected areas are outlined in grey.

The reserve network for biodiversity features is much patchier than the networks for ecosystem services (Fig. 3), as might be expected solely on the basis of biodiversity comprising hundreds of features (species, ecosystem types, etc.). The proposed “best” solution for timber production has much larger contiguous areas and is spread throughout much of the study area, with the exception of the steeper terrain in the northeast corner and the existing protected areas in the south-west (Fig. 3). The carbon storage reserve network is concentrated along the borders of the study area, away from urban areas and major highways (Fig. 3). Recreational angling is only possible in small patches dispersed across the study area; the angling reserve network follows this, with more reserves associated with clusters of small lakes along the southern border and in the center of the study area (Fig. 3).

### Efficiency

**Figure 4:**
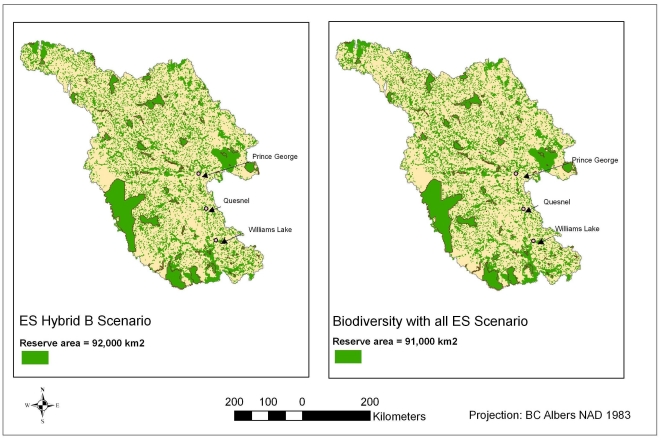
The effects of including timber opportunity costs on reserve network design. BD + ES Targeted Benefits and BD + ES Hybrid A have identical parameters except for the cost surface, which includes timber as an opportunity cost in Hybrid but not Targeted Benefits. The two Marxan-selected networks represent the same levels of recreational angling and carbon storage values (Table 2).

Fig. 4 depicts the effects of including timber harvesting opportunity costs in Marxan's cost surface (in the comparison between BD + ES Targeted and BD + ES Hybrid A—as suggested by Table 1, the two scenarios are identical except in the cost surface, where only Hybrid A includes timber production values as an opportunity cost). Hybrid A's network is dispersed across the study area with fewer compact areas outside of parks and protected areas than Features (Fig. 4). This dispersion leads to (unsurprisingly) higher ‘costs’ based on an index of road density (‘Suitability Index’ (SI)—without timber; $2.5 million, or 6.5% greater; Table 2). However, by the more appropriate measure of costs that includes the opportunity costs of foregone timber harvest, Hybrid A's cost was $18.5 billion (or 15%) less than the Targeted scenario (Table 2). This result is wholly consistent with many other studies demonstrating the efficiency gains that accompany the inclusion of more sophisticated cost data into conservation planning [31], [39].

**Table 2:** The effects of including timber opportunity costs on total costs.

Scenario	Cost (timber production and transformed road index values)	Cost (SI values)	Amount of recreational angling values captured	Amount of carbon storage values captured
BD + ES Targeted	$123.3B	38.7M	$16.9M	$10.0B
BD + ES Hybrid A	$104.8B	41.2M	$16.9M	$10.0B

BD + ES as Targeted Benefits and BD + ES Hybrid A have identical parameters except for the cost surface. The two Marxan-selected networks represent the same levels of recreational angling and carbon storage values, but they have different costs. Each performs more efficiently by the costs of its own cost surface (BD + ES Targeted Benefits was run with a cost surface including timber values, while Hybrid A was run only with the suitability index).

### Ecosystem services as targeted benefits vs. co-benefits

**Figure 5:**
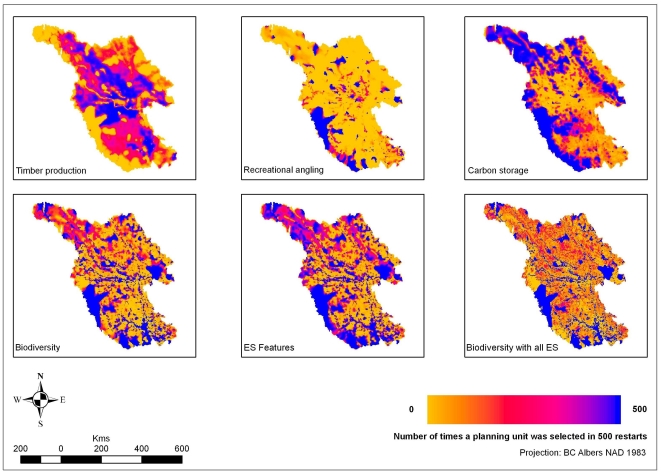
The effects of including conservation-compatible ecosystem services as targeted vs. co-benefits on reserve network design. BD + ES Co-Benefit/Cost and BD + ES Hybrid B are identical except in their treatment of recreational angling and carbon storage values (they protect the same amount of each benefit; Table 3).

Fig. 5 depicts the effects of including conservation-compatible services (recreational angling and carbon storage) as targeted benefits vs. co-benefits (in the cost surface). The resulting networks were very similar, both in their spatial distributions (Fig. 5) as well as their costs (Table 3). Treating recreational angling and carbon storage as co-benefits rather than targeted benefits resulted in a potential cost savings of $1.35 billion (1.8%; for the cost surface including all services; Table 3).

**Table 3:** The effects of including conservation-compatible ecosystem services as targeted benefits vs. co-benefits on reserve network design.

Scenario	Cost (timber production and transformed road index values)	Cost (all ecosystem services and transformed road index values)	Amount of recreational angling values captured	Amount of carbon storage values captured
BD + ES Hybrid B	$85.0B	$77.8B	$13.5M	$7.2B
BD + ES Co-Benefit/Cost	$83.7B	$76.4B	$13.5M	$7.2B

BD + ES Co-Benefit/Cost and BD + ES Hybrid B are identical except in their treatment of recreational angling and carbon storage. The two Marxan-selected networks represent the same levels of recreational angling and carbon storage values, but including these two conservation-compatible services in the cost surface yielded moderate cost savings, by either measure of costs.

### Hotspots

**Figure 6:**
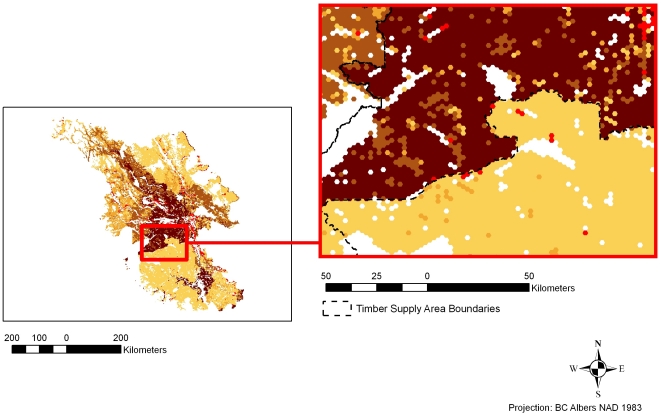
“Irreplaceability” maps for various scenarios. Each map shows the number of times each site was included in a reserve network ‘solution’ out of 500 restarts. Areas in blue could be considered hot spots for conservation efforts; areas in yellow were consistently not selected by Marxan. Areas in pink and red represent sites that were chosen in some but not all solutions.

Figure 6 provides maps of a proxy of irreplaceability [24], [40], [41], and hotspots for combinations of benefits (including services; Fig. 6). Such hotspots are areas that are consistently chosen in solutions, areas that could be considered priorities for conservation (protected areas are “locked-in” for biodiversity and conservation-compatible services, so they appear maximally hot). There are few identified hotspots for biodiversity features outside of protected areas. The hotspots for individual services are not generally consistent across services, with the partial exception of areas along the southwest border providing carbon storage and recreational angling.

Timber production represents the greatest opportunity cost to conservation in the study area. The hotspots chosen for timber are primarily in the center of the study area, close to roads as reflecting ease of transportation. These areas do not overlap with the hotspots for carbon storage. Therefore, opportunity costs related to timber production would be relatively low in the areas chosen for a carbon storage reserve network, thereby increasing the likelihood of conservation.

### Congruence of ecosystem service areas and biodiversity

Conservation-compatible services (angling and carbon storage) were positively correlated in space with biodiversity, and all these were negatively correlated spatially with timber harvest (Table 4). The correlations are not generally strong, with the strongest positive correlations occurring between the variations of reserve networks that captured biodiversity features—scenarios that had similar targets and included timber production in the cost surface.

**Table 4:** Correlations between scenarios.

Scenario	Bio-diversity	Angling	Carbon	Timber	BD + ES Targeted Benefits	BD + ES Hybrid A	BD + ES Hybrid B	BD + ES Co-Benefit	BD + ES Co-Benefit/Cost
**Biodiversity**	1.00								
**Angling**	0.19	1.00							
**Carbon**	0.23	0.20	1.00						
**Timber**	−0.14	−0.15	−0.18	1.00					
**BD + ES Targeted Benefits**	0.89	0.26	0.50	−0.21	1.00				
**BD + ES Hybrid A**	0.74	0.16	0.37	−0.27	0.78	1.00			
**BD + ES Hybrid B**	0.79	0.06	0.03	−0.09	0.64	0.82	1.00		
**BD + ES Co-Benefit**	0.81	0.06	0.08	−0.04	0.66	0.80	0.97	1.00	
**BD + ES Co-Benefit/Cost**	0.79	0.04	0.04	−0.09	0.63	0.81	0.99	0.97	1.00

The Pearson's correlation co-efficient value indicates the extent to which scenarios chose the same planning units in their solutions (1.0 indicates perfect correlation and −1.0 indicates perfect negative correlation). Values above the diagonal exclude conserved areas that were ‘locked in’ to reserve networks; values below the diagonal include these areas.

## Discussion

Our representation of ecosystem service values provides a novel and powerful approach for investigating general relationships between pairs of benefits, including ecosystem services and biodiversity, and between approaches to integrating services in conservation planning. Due to the nascent stage of research on the production and impacts to ecosystem services [42], [43], the value functions and Marxan parameters for the services contain numerous assumptions that are appropriate for these purposes but not linked sufficiently to research results for the particular context in question at finer scales. Furthermore, as previously mentioned, our analysis did not include spatially variable threats, which would certainly have influenced selected reserve networks [44], [45]. Accordingly, our results are more useful for suggesting broad patterns than for designating specific places for service protection.

### Ecosystem services as targeted benefits vs. co-benefits

In our study area, treating ecosystem services as Targeted Benefits generally yielded more spatially cohesive but costlier reserve networks than treating services as co-benefits or costs (our Co-Benefit/Cost or Hybrid approaches). While this may be due partly to the particular parameters and details of our study, there is reason to believe that the efficiency gains of the co-benefit/cost approach are general, if one assumes that the ecosystem-service valuations and their inclusion alongside other economic costs is accurate. The Co-Benefit/Cost approach represents the importance of ecosystem-service values to total costs/benefits directly, whereas the Feature approach does so through a suite of parameters (e.g., the conservation feature penalty factor (CFPF)). The purpose—and the advantage—of the Co-Benefit/Cost approach is therefore efficiency. Accordingly, if services have associated economic values (as we have argued for carbon, angling, and timber), and those values are well known, including those values in the cost surface will be the simplest means of ensuring the services are given their due weight.

Efficiency is not all-important, however, so even if economic valuations are trustworthy, the choice between the two approaches in any given context should depend largely on the nature of the benefits and values at stake. Integrating service values into the cost surface effectively treats the benefits at stake as substitutable costs or benefits that might serve instrumental roles towards conservation. Treating services as benefits for which targets should be set (and achieved) effectively treats the benefits as inherently important and not perfectly substitutable with other benefits. Whereas the former is appropriate if the principal values at stake are preference-based, as with market values, the latter is much more appropriate if the values at stake include principles and virtues [46], [47]. Accordingly, there is no single better approach to including services in planning or decision-making generally—both approaches deserve further development.

### Congruence of ecosystem service areas and biodiversity

Our study revealed a mild good-news story for conservation, in the positive correlations between conservation-compatible services and biodiversity and the negative correlations of the service considered incompatible with conservation in this this case (timber production). The spatial incongruence between carbon storage and timber production networks is an unexpected positive outcome for conservation. One might expect that areas of high timber production value would align with high carbon storage, but they did not. Many areas with high carbon storage values are characterized by variable slope, which results in high harvesting costs and thus low timber production values. Although it is important to confirm this result with more sophisticated forestry modeling, this incongruence between timber harvest values and conservation might assist conservation of biodiversity and compatible services based on relatively low opportunity costs of timber harvest.

The relatively weak and sometimes negative correlations between ecosystem services and biodiversity shown here echoes past research on ecosystem services in conservation planning [16], [17]. Obviously, protecting areas for biodiversity will not maximize provision of all services. Accordingly, there is danger in including services as priorities of conservation plans unless the manner of inclusion reflects the ways that ecosystem services can support biodiversity goals. If services have real implications for conservation costs as we assume here, including the economic values of changes in ecosystem services (from prevailing land-use to conservation) in Marxan's cost surface will appropriately skew prioritized areas toward areas of low opportunity cost and important co-benefits.

Tradeoffs are expected between certain services and biodiversity for a variety of reasons [35], [48], [49], [50], including the differing roles that roads play in producing ecosystem service benefits and affecting biodiversity conservation. For example, ecosystem services such as recreation and timber or forage production (Chan et al. 2006) rely on a proximity to roads in order to maximize their potential benefits, whereas biodiversity is frequently threatened and degraded by roads.

### Limitations of available data

**Figure 7:**
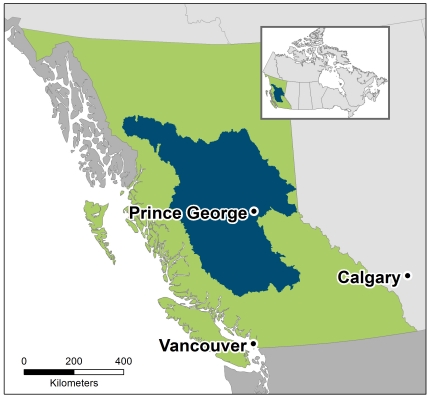
Artificial discontinuities in timber production values. These discontinuities align with timber supply area (TSA) boundaries and likely result from the use of different modeling methods amongst TSAs.

Ecosystem service research and, particularly, spatially explicit approaches to doing so are often restricted by available data. Similarly, our analysis is limited somewhat by inconsistencies in the original data. In particular, the model of timber production was based on timber supply reviews, which are reports conducted for individual timber supply areas (TSAs) in British Columbia [51]. Individual timber supply reviews use different methods to model the volume of timber at the expected minimum harvestable age. These different methods create artificial breaks between administrative boundaries in the values of timber production. The stark discontinuities in values are artifacts of the original data (Fig. 7); they underscore the importance of consistency across jurisdictions and management areas for effective conservation planning and ecosystem-based management.

### Increasing the possibility for implementation

All of the networks that used a Co-Benefit/Cost or Hybrid approach (i.e., any approach that included ecosystem services in the cost surface) consisted of many small patches that are likely not realistically implementable as conservation areas. As such, further experimentation with Marxan's parameters and connectivity analysis is needed to realize the potential of this novel approach.

The values associated with these ecosystem services are *potential*, not *realized* benefits, but the gains of including these values depend on the realization of these values. Benefits could be realized if the conservation agent (generally an NGO or government agency) were to receive any of the following: funds for carbon offsets for carbon stored on lands that would have been logged; assistance (or less opposition) from the forestry industry in exchange for bypassing conservation protection of high timber-value areas; assistance or funding from recreational angling groups for conserving areas of importance to them; or other incentives associated with new policies to internalize costs and benefits of ecosystem services. Institutional changes such as the creation of carbon storage credits and/or markets are critically enabling here. Without such policies and institutions for other ecosystem services, ecosystem-service contributions to better land-use and management decisions will be limited and dependent on individual good will and leadership.

### Conclusion

Our study both confirms previous results and offers a significant advance to the incorporation of ecosystem services into conservation planning. Our case study reveals weak positive associations between conservation-compatible services (recreational angling and carbon storage) and biodiversity, and weak negative associations of these with conservation-incompatible timber harvest. Further, it demonstrates the considerable efficiency gains of including more sophisticated cost (and co-benefit) data into planning: including services in conservation planning analyses will appropriately skew prioritization towards areas with lower costs or greater co-benefits. Our central advance is a demonstration of contrasting approaches to including services in planning: treating services as targeted benefits vs. as co-benefits/costs. In our study, the two approaches—which differ in their applicability, depending on the service—performed roughly similarly, with moderate efficiency gains for the co-benefit/cost approach.

The challenge of modeling ecosystem services is immense, but so is the opportunity: conservation projects designed and implemented effectively can benefit people and ’nature’; and rigorous analysis of ecosystem-service benefits could be instrumental in inspiring the institutional changes needed to internalize these important values in decision-making.

## Materials and Methods

The study area consists of two eco-provinces: Sub-boreal and Central Interior. Eco-provinces are regions within the province of BC that share a similar climate and topography and are also at a “reasonable” size for policy creation and implementation [52]. The topography is relatively flat in the centre, with large mountain ranges surrounding the region. Vegetation in the area is dominated by the Interior Douglas-fir and Sub-Boreal Pine-Spruce biogeoclimatic (BEC) zones and there are large areas of Bunchgrass along the valley bottoms of the Fraser River, whose headwaters are located in the Sub-boreal eco-province. The study area covers a vast landscape, roughly 46,000 km^2^ (Fig. 1), is home to a wide variety of fauna including caribou, grizzly bear, moose, mule deer, and over 65% of all bird species known in the province [52] and contains a relatively low population with the largest cities of Prince George and Quesnel totaling fewer than 90,000 residents combined.

### Economic valuation of ecosystem services and mapping of services and biodiversity features

From the beginning, we assumed—following the larger ecoregional assessment led by NCC—that timber harvesting would be the greatest direct threat to services and biodiversity and would present the most valuable opportunity cost from conservation. Thus, we set out to measure the differences in economic values of ecosystem service provision under two land-use scenarios: conservation and timber harvesting. Three services were employed for valuation: carbon storage, timber production, and the provision of recreational angling opportunities (see Fig. 7). These were then integrated into a further analysis based on three different approaches: Targeted Benefit, Co-Benefit/Cost, and a Hybrid approach. All services were valued and mapped in 500-hectare planning units that were later included in the conservation planning exercise using the software program Marxan. Below we present our assumptions and briefly summarize the methods used to model and value each service. For greater detail, see Appendix A (Text S1).

### Carbon storage

To calculate long-term biophysical carbon storage capacity, we combined a very coarse (∼9×9 km) static assessment of current carbon storage with a model representing how carbon storage varies across time in a given forest landscape. For the former, we used publicly available data from the World Resources Institute containing information about carbon storage in soil, as well as above and below ground vegetation [34]. We assumed that variation in these data correlate with variation in carbon storage capacity—effectively assuming that each 9×9 km quadrant has been logged to similar degrees. (While this assumption may be incorrect, the carbon budget modeling suggests that timber harvest has a relatively small impact on standing carbon at such coarse scales—see below; accordingly, violations of the assumption should have small effect on the results.) For the latter, we used information derived from the Carbon Budget Model of the Canadian Forest Service to calculate the long-term depression of carbon storage as a result of a harvest cycle. The Canadian Forest Service's model has been used to measure differences in carbon storage across land uses in a forested landscape similar to our study area [53].

This model was used to determine the change in carbon storage in two hypothetical BC Interior forest landscapes that differed only by their fire disturbance and managed harvest cycles. The landscapes had fire disturbance cycles of 500 and 750 years and harvesting cycles of 100 and 120 years respectively. To calculate carbon loss we adopted findings from Kurz et al. [53] who found an 18.2% and 1% loss, respectively, in carbon when the landscapes transitioned from a “natural” to a “managed” management scenario. We took the rounded average of these findings and assumed a 9.6% loss of carbon when an area was logged versus when it was conserved. These figures assume sustainably managed forest practices, as well as regular fire and pest disturbances. So, if all land is currently being managed, it is at 90.4% of its carbon storage potential and has 10.6% to gain if it were conserved (in proportional terms: 1 = 0.90.4 * 1.106; we get 10.6% from 1.106 - 1). Conversely, if all land is currently conserved, then it may lose 9.6% of its carbon if it were harvested. Therefore, we valued and mapped 10% of the carbon storage values in the study area as a rounded estimate averaging across land that is currently not being harvested (the difference between land-uses is 9.6% of current carbon storage) and that which is (difference is 10.6%). Since specific locations of cut-blocks are not publicly available, some assumptions were made about the amount and location of possible harvests.

Our economic values for carbon storage reflect avoided social damages associated with climate change. We valued carbon storage at $8.46 (CDN) per ton of carbon dioxide using the mid-price average of three carbon trading markets: the Chicago Climate Exchange, the New South Wales and the EU Emissions Trading scheme on March 19th, 2008. We followed the methods and assumptions outlined by Naidoo and Ricketts [54] to justify the use of carbon credit trading prices as proxies for the value of carbon storage. There are two assumptions related to this calculation. First, we assumed that the beneficiaries of this ecosystem service are global and that these prices reflect the amount of social damage avoided to society at large by decreasing CO2 emissions [54]. Second, we assumed that protection against deforestation is a valid strategy to reduce CO2 emissions and that those areas inside of the study area are under imminent threat of deforestation. The high levels of logging activity in the area support this assumption.

The coarse resolution of the data hides variations within each cell; thus carbon values represent averages across individual stands at different ages/stages, with a distribution of stages resulting from the historic management regime. We assumed that each cell has been managed according to a sustainable yield model for harvest rotations specified by its particular TSA, such that for cells that are managed with a 40-year rotation approximately 1/40th of the harvestable area in each cell was harvested each year for the past 40 years. Accordingly, we may slightly overestimate or underestimate the change in carbon storage in any cell based on whether harvest was more or less recent than half of the listed rotation length. Subject to these assumptions, our value represents the net present value (NPV) of social damage avoided by the difference in carbon storage associated with timber harvesting/conservation.

### Timber production

Timber production is measured here as an opportunity cost of conservation, with the difference between the two land-use scenarios being 100% of the net value of timber harvest. NCC does not intend to allow regular timber harvest within reserves.

We calculated NPVs for timber production over a 1000-year timeframe with a discount rate of 4%, assuming a constant ratio of benefits to costs, based on expert opinion and forestry economics literature [55]; Nelson pers. comm.]. Although this long timeframe differs from the 25-year timeframe for recreational angling, 25-year NPVs would be only 36% less due to the small contribution of years in the distant future. All values were measured per 500-ha planning unit and assume uniform costs and benefits within each cell. We believe this simplification is necessary given the hundreds of thousands of planning units within this large study area.

Costs consisted of harvesting costs, cost of transportation to the closest processing facility, and the costs of replanting (silviculture costs). These costs were based on slope, distance and biogeoclimatic zone, respectively, and were derived from previous merchantability assessments in the province [38]. Steeper slopes and longer transport distances result in higher costs. Biogeoclimatic zones are used by the Interior Appraisal Manual to distinguish between different silviculture costs in the province [56]. The benefits of timber production were measured as a function of leading tree species and the volume expected at its minimum harvestable age. Average timber prices were calculated from BC Interior Log Market Reports from 2003–2008 [57].

### Recreational angling

We assumed that timber harvesting and associated activities will have an adverse effect on recreational angling values through an increase in sedimentation [58], [59], and that recreational angling activities are consistent with conservation. Such an assumption is only appropriate at the coarse resolution of our illustrative analysis, as particular effects of forestry operations on fish populations are highly variable and contingent upon both context and management details [37].

We determined the value of recreational angling in the study area, and how much it may be impacted by timber harvesting activities, using data from an angler effort model that predicts how much actual angler effort (measured in days spent fishing) can be supported by a particular lake given its productivity, distance from major population centers, and accessibility by roads. Parkinson et al. [60] fitted the model using raw data such as boat counts from aerial surveys as well as mail surveys in the region. We matched the number of angling days for each lake with economic values for the average amount of money spent per day on recreational angling in freshwater regions of BC, which includes transportation as well as licenses, package deals and accommodation [61]. We translated yearly values into NPVs for a 25-year timeframe and a 4% discount rate.

Using the Ministry of Environment's Fisheries Sensitive Watershed database, we assigned relative sensitivity scores (from 0 to 1.0) to third-order watersheds in the study area based on equal weighting of six characteristics: soil type, density of alluvial streams, lake buffering capacity, amount of forest cover, annual precipitation, and slope [62]. These data were only available for catchments that contribute to smaller lakes, ones assumed to be not artificially stocked. Given our objectives of representing the value of angling at risk due to sedimentation, it is appropriate to exclude stocked lakes because frequent stocking buffers fish populations from the ill effects of sedimentation.

For simplicity in the absence of other understanding, we assumed a linear 1∶1 relation between sensitivity to timber harvesting and change in economic values of recreational angling. Thus we combined the sensitivity score of each watershed with its recreational angling value, based on amount of effort, to derive a final value of the expected difference in recreational angling values between conservation and timber harvest scenarios (e.g., a watershed with a sensitivity score of 0.10 and potential economic value of $10,000.00, would be assigned a value-difference of $1000.00 attributable to recreational angling, between timber harvest and conservation land-uses).

### Terrestrial biodiversity

Biodiversity features were divided into coarse and fine filter features such as old growth forest ecosystems and rare plant species, respectively. The fine filter data consist of over 75 plant species and 100 animal species (3 amphibians, 5 reptiles, 28 mammals and 64 birds). Animal species were selected based on their designation as threatened on provincial, national, and international lists, as well as other more expert-informed subjective characteristics such as whether the species is endemic, regionally important or especially vulnerable to change. Data used to represent these features came from a variety of sources including the BC Conservation Data Centre, the BC Ministry of Environment, the Canadian Wildlife Service and Ducks Unlimited.

The data represent terrestrial ecological systems, as defined by the NatureServe classification system [63]. These systems are meant to represent groups of biological communities that are found in similar physical environments and are influenced by similar dynamic ecological processes, such as fire or flooding. Examples of such systems include the North Pacific Interior dry grassland and the North Pacific Mountain Hemlock Forest. Coarse filter data also included particular rare or “focal” ecosystems, such as hot springs and stands of old growth forests.

### Inclusion of ecosystem services values in Marxan

#### Marxan scenarios

We used the site selection program Marxan (version 2.0.2) to select reserve networks [24]. Marxan is an algorithm for achieving stated conservation goals while minimizing costs, where those costs are minimized through ‘simulated annealing’ pseudo-optimization, which is appropriate in light of the complexity of the problem (involving hundreds to thousands of features and many thousands of spatial planning units) [24], [64]. Its objective function includes a cost surface and two kinds of penalties: the CFPF, for failing to achieve targets, and the boundary length modifier, for reserve perimeter. Except where noted in Appendix B (Text S1), the Marxan Good Practices Handbook [65] was used to establish the parameters for the planning scenarios.

Our results focused on the “best” and “summed solutions” outputs. The “best” solution from each scenario is the reserve network that has the lowest objective function score and meets all targets [24], and the “summed solution” is the mapped values representing the number of times (of 500 restarts) a particular planning unit is included in a final solution, which indicates how important a particular planning unit is to the reserve network—its irreplaceability. Each scenario, the approach, features used and the associated cost surface(s) are given in Table 1 (for additional details regarding the parameters for each scenario, see Appendix B (Text S1)).

We did not incorporate variable threats (probability of habitat loss) but rather assumed that all areas not protected would eventually be subject to degradation (the ‘scorched-Earth’ approach). This was in line with the approach of the NCC and their ecoregional assessment. While we see great value in incorporating a threats analysis into conservation planning, there was no available defensible threats analysis for this study region.

Wherever possible, we maintained parameter values as closely as possible across scenarios. In some cases, this may seem odd (e.g., why have a CFPF for services?), but it is important for two reasons. First, the context here is of conservation, and if ecosystem services are to be helpful for biodiversity protection, the management needs and costs will parallel those of biodiversity, to a degree. Second, maintaining consistency of parameters across scenarios is key for maintaining comparability across scenarios.

#### Targets for ecosystem services: the Targeted Benefit approach

Ecosystem services have generally been included in conservation assessments as benefits for which particular targets are desired [65], although the specified benefits have frequently been proxies of services, such as ecosystem processes and biodiversity patterns [20]. We ran Marxan in multiple scenarios with ecosystem services as benefits and assigned targets for each. These targets required Marxan to include at least 50% of the total available ecosystem service values within each solution. There is inherent difficulty in choosing meaningful targets for services. We chose 50% in order to maintain flexibility within Marxan solutions (i.e., 100% solutions would select all available areas) but also to represent a large portion of each benefit. Sensitivity analysis on the targets (with targets of 40% and 60% of the total values) revealed only minor changes in the spatial correlations between scenarios. Accordingly, 50% targets were used in all of the scenarios except in ES Hybrid B, which was created solely for comparison with BD + ES Co-Benefit/Cost (thus, we used the values of recreational angling and carbon storage captured in the latter scenario as their respective targets in ES Hybrid B).

#### Ecosystem services in the suitability index: The Co-Benefit/Cost approach

We ran separate scenarios in Marxan that included carbon storage, recreational angling and timber production ecosystem service values in the suitability index (using the transformation explained below). By associating ecosystem service values as co-benefits (for angling and carbon) or costs (for timber) with each planning unit, we effectively increase or decrease the cost of including the planning unit in the reserve. Despite calls to do so, researchers have rarely included socio-economic values in choosing areas for conservation [but see 30,31,54,66]; this novel Benefit/Cost approach offers a simple means for conservation planning to better reflect implications for human well-being.

To integrate ecosystem-service values into the SI, we had to convert the SI road index scores into dollar values reflecting fee-simple acquisition costs. NGOs including NCC also ‘acquire’ land through conservation easements [67], and acquisition costs would be lower for easements, but easement agreements generally entail additional transaction and monitoring costs. These additional costs also might be expected to scale with road density, since road density was chosen for a suitability index in part because higher road density generally entails a greater diversity and magnitude of threats to be managed. Accordingly, we adopt land prices as an imperfect but defensible proxy for costs.

For the conversion to dollar values we used a four-part linear transformation based on land prices (acquisition costs) in the study area, assuming that higher SI scores (higher road density or proximity to roads) correlate with higher land prices due to urbanization. Because NGOs also receive land donations, the market price may be an overestimate of acquisition costs, possibly rendering our transformation to market prices somewhat conservative. But it is impossible to account for the unpredictability and site-specificity of donations at an ecoregional scale. For greater detail on the SI and our transformation, please see Appendix C (Text S1).

We then added timber production values and/or subtracted recreational angling and carbon storage values from the transformed SI. We thereby assume that ecosystem services increase (in the case of timber production) and/or decrease (in the case of recreational angling and carbon storage) the costs or difficulty of conservation. For example, an area with high carbon storage values may be more easily conserved as some of the costs of conservation might be recouped through future fiscal returns via carbon credits. In a similar way, an area with high timber production values may be found to have opportunity costs that render conservation socially unacceptable.

We compared the efficiency of this Co-Benefit/Cost approach to the Targeted Benefit approach by comparing the cost of the “best” solutions in the two approaches [65]. Because the scenarios were run with different cost surfaces in Marxan, this required *ad hoc* calculations of total costs, to ensure fair comparisons. First we identified the areas in the “best” reserve network for biodiversity, recreational angling and carbon storage benefits together (our Targeted approach). We then determined the cost of this reserve network inclusive of ecosystem service values (along with transformed road index costs)—although the cost surface of Marxan in this case had not included the service values. We compared this summed cost to the cost of a reserve network that protected biodiversity but included recreational angling, carbon storage and/or timber production values within the cost surface (our Co-Benefit/Cost approach).

#### Spatial congruence

In order to assess the spatial correlation between “summed solutions” for each scenario we calculated Pearson's correlation coefficients for each pair of scenarios (Table 4). These values indicate similarity between individual networks.

## Supporting Information

Text S1 **Three appendices providing additional details on methods.** Appendix A (data sources and methods for ecosystem service modeling and valuation); Appendix B (details of Marxan scenarios); and Appendix C (suitability index transformation).
(DOCX)
