# Supplementary material for: Ecosystem Services in Conservation Planning: Targeted Benefits vs. Co-Benefits or Costs?
Source: PLoS One. 2011 Sep 6;6(9):e24378. doi: 10.1371/journal.pone.0024378 (PMC3167855; doi:10.1371/journal.pone.0024378)
Supplement: Text S1 — Three appendices providing additional details on methods. Appendix A (data sources and methods for ecosystem service modeling and valuation); Appendix B (details of Marxan scenarios); and Appendix C (suitability index transformation). (DOCX) [file pone.0024378.s001.docx]

**Ecosystem Services in Conservation Planning: Targeted Benefits vs. Co-benefits or Costs?**

Dr. Kai M.A. Chan: Institute for Resources, Environment and Sustainability, University of British Columbia, Aquatic Ecosystems Laboratory, 438-2202 Main Mall, Vancouver BC Canada, V6T 1Z4

Lara Hoshizaki: Institute for Resources, Environment and Sustainability, University of British Columbia, Aquatic Ecosystems Laboratory, 2202 Main Mall, Vancouver BC Canada, V6T 1Z4

Dr. Brian Klinkenberg: Department of Geography, University of British Columbia, 1984 West Mall, Vancouver BC Canada, V6T 1Z2

##

# Supporting Information S1

## Table of Contents

Appendix A: Data sources and methods for ecosystem service modeling and valuation

Appendix B: Details of Marxan scenarios

Appendix C: Suitability index transformation

References

# Appendix A: Data sources and methods for ecosystem service modeling and valuation

## Carbon storage

The World Resources Institute provided a dataset for carbon storage—in tons/ha—in above and below ground vegetation and soil, in a grid with 82.81km² cells [1,2]. The cells were disaggregated to 500ha hexagons using area-weighted averages.

To determine the difference in carbon storage between an actively forested landscape and a conserved landscape, we conducted a literature review of carbon storage changes in landscape transitions. Kurz et al. [3] provided a relevant case study as a basis for our work as they modeled carbon changes between a managed and natural forest in the Interior region of British Columbia. This study reported a 10% decrease in stored carbon when an area transitioned from a natural to sustainably harvested state. Their model also accounted for losses in carbon due to natural disturbances, such as fire and pests. Similar losses were also found by [4], and Leighty et al. [5].

Therefore, we assumed that the difference in stored carbon between a conserved and harvested landscape was 10%. However, there was no available data to tell us what areas of the landscape were currently being harvested and which were in a natural state. Therefore, if we assume that the entire landscape is currently in a natural state, that is, at 100% of possible carbon storage potential, the difference between conservation and forestry will be a 9.6% loss. However, if we assume that the entire landscape is being harvested, that is, at 90.4% of possible carbon potential, the potential gain in carbon storage would be 10.6%. To determine the average difference in carbon storage between the two scenarios, we used the intermediate rounded figure, 10%. Therefore, the benefit function to describe the change in carbon storage between a conserved and forested landscape was as follows:

∆Carbon Storage = 0.1 x Current carbon storage

CO2 storage was valued at $8.46 (CDN) per ton using the mid-price average of three carbon trading markets: the Chicago Climate Exchange, the New South Wales and the EU Emissions Trading scheme on March 19^th^, 2008. This value was then multiplied by 500 and assigned to individual hexagons in the study area.

## Timber production

Timber production was considered as an opportunity cost of conservation, therefore the difference in production values is effectively 100%. These values were measured in a way that considered both the merchantability of the timber within a particular hexagon, as well as the net present value of that timber.

To measure timber production value across the landscape, a spatially-explicit database was created using a variety of sources (Table A1). Hexagons were removed from the database if they were considered to be outside of any possible timber harvesting land base (THLB). Because the THLB changes over time, we assumed hexagons will not be harvested if their centroid was within a land use that would not be suitable for timber harvesting (alpine and tundra BEC zone; bunch grass BEC zone; urban; lakes; shrub – from baseline thematic mapping; range – from baseline thematic mapping; parks and protected areas). For each remaining hexagon within the THLB, an average leading species was determined using Vegetation Resource Inventory (VRI) raster data and zonal statistics in ArcGIS 9. The economic values assigned to the hexagon were based on this leading species and assume that the species that covers the majority of the hexagon is a representation of the range of values present within the hexagon.

**Table A1**: Spatial data sources for timber production

| **Data** | **Resolution** | **Source** |
| --- | --- | --- |
| Leading species | 1 ha | Vegetation Resources Inventory via HectaresBC |
| Site index | 1 ha | Vegetation Resources Inventory via HectaresBC |
| Leading age | 1 ha | Vegetation Resources Inventory via HectaresBC |
| Biogeoclimatic zones | 1:20, 000 | Ministry of Forests and Range |
| Slope | 25m | Nature Conservancy of Canada |
| Mill locations | unknown | Ministry of Forests and Range |

Each hexagon was assigned a leading species, an average site index of that species and the Timber Supply Area (TSA) number that it was in. Using this information we reviewed the Timber Supply Review (TSR) Analysis report for each hexagon and determined the minimum harvestable age (MHA) and volume of harvestable timber present at that age. The MHA is the earliest age at which the timber could be harvested, and using this value offers a conservative estimate of volume and market value. The leading species and volume at MHA determined the net benefit of each hexagon based on average prices from the 2003 – 2008 BC Interior Log Market reports [6].

Costs were calculated as a summation of harvesting, transportation and silviculture costs. Harvesting costs were based on the average slope of the hexagon and transportation costs were based on the distance of each hexagon to the nearest primary processing facility using the spatial join function in Arc. Both costs were measured using an index from a similar merchantability assessment done in the Interior of BC [7] and were assumed and confirmed to be appropriate by experts.

A loop function was written in Visual Basic to determine the NPV of each hexagon given its total value as well as the number of harvesting rotations expected within a given time period (see below). The costs and benefits of each hexagon were assumed to be constant throughout the time frame.

The number of harvests, or rotations, within the time period was determined by the minimum harvestable age of the stand, as well as its current age. If the average current age of the hexagon was greater or equal to its MHA, it was assumed that the hexagon would be harvested immediately. If the stand’s age was less than the MHA, the function used the difference in years between the current age and the MHA to account for the time before the initial harvest. After determining the number of rotations, the NPV was calculated using a time frame of 1000 years and a discount rate of 4%. Both of these figures are commonly used in forestry value assessments [8,9,10].

### Visual Basic script to calculate net present value (NPV):

'B is benefits--assumed to be constant

'C is costs--assumed to be constant

Function NPV_tree(B, C, r, Tp, MHA, age)

If (age >= MHA) Then

init_harv = 0 'If the current age of the stand is greater or equal to the MHA then there will be a harvest immediately.

'Rot is the # of rotations

Rot = Round((Tp / MHA) + 0.5) ' If the current age is greater or equal to the MHA, then the number of rotations will be the time period (1000)/MHA plus 1 (for the immediate harvest). Adding .5 and rounding effectively adds one harvest rotation.

Else

Rot = Round((Tp - (MHA - age)) / MHA) + 0.5 ' If the current age is less than the MHA then the number of rotations will be the time period minus the difference between the MHA and current age divided by the MHA. This will be rounded up to account for the time spent reaching the initial harvest.

init_harv = MHA - age 'If the stand's age is less than the MHA, the initial harvest will occur once the stand has reached the MHA

End If

ti = init_harv ' Time when harvesting begins

Sum = 0

Dim i As Integer 'declares i as an integer

For i = 0 To (Rot - 1) 'for loop function

Sum = Sum + (B - C) / ((1 + r) ^ ti) ' Summation of NPV function

ti = ti + MHA

Next i

NPV_tree = Sum

## Recreational angling

The expected decrease in the economic values of recreational angling in the study area due to timber harvesting was measured using two separate models.

Angler effort, or the amount of angling days that would be supported by a particular lake, was modeled previously by Eric Parkinson [11]. These data were only available for non-stocked lakes; however, this was not seen as a limitation as the amount of angling available in artificially stocked lakes is assumed to be independent of surrounding timber harvesting. Effort was modeled as a function of lake productivity, distance from major population centers and proximity to roads. The amount of effort per lake was converted into dollar values by dividing the total amount of days fished by the amount of revenue generated in that year. This figure includes, but is not limited to, money spent on transportation, licenses, equipment and package tours. These revenue statistics are only relevant for freshwater regions in British Columbia and are derived from the Survey of Recreational Fishing in Canada [12].

The second set of modeled data was used to classify each 3^rd^ order watershed in the study area with a relative sensitivity to timber harvesting score. In general, when we discuss sensitivity we are referring to the likelihood of increased sedimentation within streams. To rank this sensitivity, we used data from the Fisheries Sensitive Watershed (FSW) database that evaluates watersheds in British Columbia that may be particularly susceptible to a decrease in fisheries due to logging [13]. The variables included in the FSW index are: soil type, annual precipitation, lake buffering capacity, amount of area with gradients over 60%, amount of forest land cover, and the density of alluvial channels. For example, a watershed with highly erodible soil, heavy annual precipitation, low lake buffering capacity, high slopes, large amount of available timber and a high density of alluvial channels would be considered highly sensitive to logging.

Using a GIS, these sensitivity variables were combined with equal weighting within a multi-criteria evaluation. There is current debate regarding the combination of these different variables; however, at the time of writing equal weighting is common practice. All watersheds were then given a relative ranking from 0 -1.0. The amount of effort was assigned to the entire watershed that the lake was present in in order to create a continuous surface and scale of both effort and sensitivity.

To disaggregate the individual 3^rd^ order watersheds into 500ha planning units for a Marxan analysis, we assumed that steep planning units will contribute to economic losses more than flat ones. However, all planning units will contribute to an area’s overall sensitivity. Given this, we assigned individual values to planning units using the equation:

l = (L/N)*((s+x)/(S+x))

Where l = economic loss attributed to individual planning unit

L = total economic losses in watershed

N = number of planning units in watershed

s = average slope of individual planning unit

x = 49% (largest planning unit slope)

S = average slope of watershed

# Appendix B: Details of Marxan scenarios

Due to the high number of biodiversity features included in the analysis, we found through testing that the value of the boundary length multiplier (BLM) would have to be extremely large in order to achieve spatial cohesion in any of the solutions. Under the direction of the NCC, we used a BLM of 1 in all runs in order to utilize the penalty, but ensure that costs to the reserve network were not astronomical. We used conservation feature penalty factor (CFPF) of 10 for all targets as it was the lowest value found that would still ensure all targets were met. Scenarios were run separately for each eco-province due to the large size of the study area and the amount of data associated with it, but we combined the results for a Pearson’s correlation analysis of spatial congruence across scenarios. (Although our data do not follow a normal distribution, we present these results for descriptive purposes only, not significance testing.)

As a proxy for costs—in concordance with the NCC biodiversity assessment—we used a ‘suitability index’ (SI) that assigns a non-monetary cost value to each planning unit based on its density of and/or proximity to roads (Table B1). The SI was created by the Nature Conservancy of Canada to indicate (un)suitability of a particular planning unit for conservation. The index assigns a relative score to each planning unit where higher scores indicate greater density or proximity to roads, which incur greater costs of conservation and greater vulnerability of targeted features. A “flat” cost surface was also used to assign a value of 500 to each planning unit as each unit covered an area of 500 hectares. Although area is generally a poor proxy for costs [14], it is often used as such in Marxan [15]. We used the flat surface to select areas for their high timber production values (costs are incorporated in the Benefit/Cost function), thereby creating a reserve network for activities contrary to the goals of conservation [similar to 16]—in effect a timber reserve.

**Table B1**: Suitability indices associated with Marxan scenarios.

| **Suitability index** | **Features** | | | | |
| --- | --- | --- | --- | --- | --- |
|  | **Biodiversity** | **Recreation** | **Carbon storage** | **Timber production** | **Rec., Carbon, & Bio.** |
| Road Index | x | x | x |  | x |
| Road index including carbon storage and recreational angling | x |  |  |  |  |
| Flat SI = 500ha (area of planning unit) |  |  |  | x |  |

We preselected (“locked in”) current protected areas and parks in each scenario except the timber reserve (which excluded these areas).

The exact targets in Marxan and their rationales are displayed in Table B2.

**Table B2**: Targets for individual ecosystem services

| **Feature** | **Conservation Goal Unit** | **Standard Targets** | **Percentage of total value in study area** | **Rationale** |
| --- | --- | --- | --- | --- |
| Timber production | CDN Dollar | $35.6 B | 75% | Goals relate to the creation of a timber reserve. This target is not congruent with biodiversity goals and should not be run with biodiversity features, or other ecosystem services. |
| Carbon storage | CDN Dollar | $10.0 B | 50% | Precise targets for market-based ecosystem services are difficult to assign because of the unpredictability of markets. Also, there is no ethical imperative to ensure a minimum representation of market-based services through the use of goals. In the face of this uncertainty, we have suggested general target scenarios. |
| Recreational Angling | CDN Dollar | $169.9 M | 50% | See above: Carbon storage. |

Further details on the scenarios can be found in Tables B3a and B3b.

**Table B3a**: Details of Marxan scenarios for Sub-boreal eco-province^[[1]](#footnote-1)^

| **Scenario** | **Features** | **SI** | **BLM** | **CFPF** | **Avg. Score** | **Avg. Cost** | **Avg. Boundary Length** |
| --- | --- | --- | --- | --- | --- | --- | --- |
| **Biodiversity** | Biodiversity | Road index | 1 | 10 | 3.40E+07 | 1.53E+07 | 1.85E+07 |
| **Angling** | Recreational Angling (RA) | Road index | 1 | 10 | 9.11E+06 | 3.48E+06 | 5.64E+06 |
| **Carbon** | Carbon Storage (C) | Road index | 1 | 10 | 2.35E+07 | 1.06E+07 | 1.29E+07 |
| **Timber** | Timber Production (TP) | Flat (500 per hexagon) | 1 | 10 | 1.50E+07 | 6.63E+06 | 8.34E+06 |
| **BD + ES Targeted Benefits** | Biodiversity, RA and C | Road index | 1 | 10 | 3.62E+07 | 1.72E+07 | 1.86E+07 |
| **BD + ES Targeted Benefits** | Biodiversity, RA and C | Road index and TP | 1 | 10 | 5.32E+10 | 5.07E+10 | 4.68E+07 |
| **BD + ES Hybrid (A & B)** | Biodiversity, RA and C | Road index and TP | 1 | 10 | 4.16E+10 | 3.90E+10 | 3.87E+07 |
| **BD + ES Co-Benefits** | Biodiversity | Road index, RA and C | 1 | 10 | 2.97E+10 | 2.67E+10 | 3.75E+07 |
| **BD + ES Co-Benefits/ Costs** | Biodiversity | Road index, RA, C and TP | 1 | 10 | 3.77E+10 | 3.46E+10 | 3.82E+07 |

**Table B3b**: Details of Marxan scenarios for Central Interior eco-province

| **Scenario** | **Features** | **SI** | **BLM** | **CFPF** | **Avg. Score** | **Avg. Cost** | **Avg. Boundary Length** |
| --- | --- | --- | --- | --- | --- | --- | --- |
| **Biodiversity** | Biodiversity | Road index | 1 | 10 | 3.53E+07 | 1.92E+07 | 1.60E+07 |
| **Angling** | Recreational Angling (RA) | Road index | 1 | 10 | 1.25E+07 | 5.57E+06 | 6.90E+06 |
| **Carbon** | Carbon Storage (C) | Road index | 1 | 10 | 2.91E+07 | 1.72E+07 | 1.18E+07 |
| **Timber** | Timber Production (TP) | Flat (500 per hexagon) | 1 | 10 | 1.13E+07 | 5.42E+06 | 5.91E+06 |
| **BD + ES Targeted Benefits** | Biodiversity, RA and C | Road index | 1 | 10 | 3.77E+07 | 2.22E+07 | 1.54E+07 |
| **BD + ES Targeted Benefits** | Biodiversity, RA and C | Road index and TP | 1 | 10 | 5.63E+10 | 5.53E+10 | 3.29E+07 |
| **BD + ES Hybrid (A & B)** | Biodiversity, RA and C | Road index and TP | 1 | 10 | 4.78E+10 | 4.67E+10 | 2.70E+07 |
| **BD + ES Co-Benefits** | Biodiversity | Road index, RA and C | 1 | 10 | 3.79E+10 | 3.70E+10 | 2.63E+07 |
| **BD + ES Co-Benefits/ Costs** | Biodiversity | Road index, RA, C and TP | 1 | 10 | 4.35E+10 | 4.25E+10 | 2.67E+07 |

# Appendix C: Suitability index transformation

The road index, or suitability index, was used as a cost layer in some Marxan runs (Table C1). This layer was used to assign the road index scores—which ranged between 0.0086 and 0.73 and were used as a proxy for cost—to all planning units in the study area. It represents the assumption that the cost of conservation in a planning unit is high if that unit has high road density and/or is in close proximity to roads. In particular Marxan runs, we also included ecosystem service values within the suitability index. To do this, we transformed the road index value to a dollar value that we could then add or subtract our ecosystem service dollar values to and create a cost layer that considers both roads as well as ecosystem services.

**Table C1**: Marxan run scenario descriptions and names. BD = biodiversity; ES = ecosystem services. Hybrid A uses the same targets for ES features as all other scenarios, whereas Hybrid B uses the amount of ES captured in the BD + All ES Benefit/Cost scenario as targets for the ES features.

| **Scenario** | **Approach** | **Features** | **Cost Surface** |
| --- | --- | --- | --- |
| **Biodiversity** | Targeted benefit | Biodiversity | SI – road index |
| **Angling** | Targeted benefit | Recreational angling | SI - road index |
| **Carbon** | Targeted benefit | Carbon storage | SI - road index |
| **Timber** | Targeted benefit | Timber production | Flat |
| **BD + ES Targeted Benefits** | Targeted benefit | Biodiversity and compatible ES (recreational angling and carbon storage) | SI - road index |
| **BD + ES Hybrid (A & B)** | Hybrid | Biodiversity and compatible ES (recreational angling and carbon storage) | Transformed road index and  incompatible ecosystem services (timber production) |
| **BD + ES Co-Benefits** | Co-Benefit/Cost | Biodiversity | Transformed road index with recreational angling and carbon storage |
| **BD + ES Co-Benefits/**  **Costs** | Co-Benefit/Cost | Biodiversity | Transformed road index with recreational angling, carbon storage and timber production |

To transform the road index into dollar values, we used land market acquisition values in the study region as a substitute, effectively assuming (1) that with increased road density or proximity to roads, we can expect greater urbanization and an increase in land acquisition costs; (2) that land acquisition costs are an important contributor to, and an appropriate proxy for, total costs; and (3) that land acquisition costs are over-represented by land prices to the same degree that total costs are greater than land acquisition costs. Land costs for rural areas were provided by the Nature Conservancy of Canada and costs for urban areas were found on the Multiple Listing Service (MLS) of the Canadian Real Estate Association.

We used a four part linear transformation to assign dollar values to each planning unit, based on four land values, which were placed at frequency peaks in the distribution of road index values (Table C2). We used the equation y = mx + b, according to the following:

y = Land acquisition cost

x = Road index score

m = (y² - y¹) / (x² - x¹)

b = y¹ - m¹*x¹

**Table C2**: Values used in linear transformation of Road Index (SI) to dollar values using local land acquisition costs ($)

| **$/Planning unit** | **SI** | **y1** | **y2** | **x1** | **x2** | **m** | **b** |
| --- | --- | --- | --- | --- | --- | --- | --- |
| 617750 | 0.0086 | 617750 | 1482600 | 0.0086 | 0.017 | 102958333.33 | -267691.66 |
| 1482600 | 0.017 | 1482600 | 4942000 | 0.017 | 0.35 | 10388588.59 | 1305993.99 |
| 4942000 | 0.35 | 4942000 | 7536550 | 0.35 | 0.5 | 17297000.00 | -1111950.00 |
| 7536550 | 0.5 | 7536550 | 1606150000 | 0.5 | 0.7 | 7993067250.00 | -3988997075 |
| 1606150000 | 0.7 |  |  |  |  |  |  |

# References

1. Matthews E, Payne R, Rohweder M, Murray S (2000) Pilot analysis of global ecosystems: Forest ecosystems. Washington, DC: World Resources Institute.

2. Loveland TR, Reed BC, Brown JF, Ohlen DO, Zhu Z, et al. (2000) Development of a global land cover characteristics database and IGBP DISCover from 1 km AVHRR data. International Journal of Remote Sensing 21: 1303-1330.

3. Kurz WA, Beukema SJ, Apps MJ (1997) Carbon budget implications of the transition from natural to managed disturbance regimes in forest landscapes. Mitigation and Adaptation Strategies for Global Change 2: 405-421.

4. Sanscrainte CL, Peterson DL, McKay S (2003) Carbon storage and soil properties in late-successional and second-growth subalpine forests in the North Cascade Range, Washington. Northwest Science 77: 297-307.

5. Leighty WW, Hamburg SP, Caouette J (2006) Effects of management on carbon sequestration in forest biomass in southeast Alaska. Ecosystems 9: 1051-1065.

6. Ministry of Forests and Range (2010) Log Market Reports.

7. Thomae O (2008) East Kootenay Timber Merchantabilty Analysis. 20 p.

8. Alaouze CM (2004) The effect of conservation value on the optimal forest rotation. Land Economics 80: 209-223.

9. Creedy J, Wurzbacher AD (2001) The economic value of a forested catchment with timber, water and carbon sequestration benefits. Ecological Economics 38: 71.

10. Heal G (2000) Nature and the Marketplace: Capturing the Value of Ecosystem Services. Washington, D.C.: Island Press. 203 p.

11. Parkinson EA, Post JR, Cox SP (2004) Linking the dynamics of harvest effort to recruitment dynamics in a multistock, spatially structured fishery. Canadian Journal of Fisheries and Aquatic Sciences 61: 1658.

12. Government of Canada FAOSS (2009) 2005 Survey of Recreational Fishing in Canada. Fisheries and Oceans Canada.

13. Reese-Hansen L, Parkinson EA (2006) Evaluating and designating Fisheries Sensitive Watersheds: An overview of BC‚Äôs new FSW procedure. BC Ministry of Environment. 46 p.

14. Naidoo R, Balmford A, Ferraro PJ, Polasky S, Ricketts TH, et al. (2006) Integrating economic costs into conservation planning. Trends in Ecology & Evolution 21: 681-687.

15. Ardron JA, Possingham, H.P., and Klein, C.J. (2008) Marxan Good Practices Handbook. Vancouver, BC. 155 p.

16. Ban NC, Vincent ACJ (2009) Beyond marine reserves: exploring the approach of selecting areas where fishing is permitted, rather than prohibited. PLoS ONE 4: e6258.

1. The values for the boundary length modifier (BLM) and conservation feature penalty factor (CFPF) were determined by the Nature Conservancy of Canada. [↑](#footnote-ref-1)
